# Navigating the Intricacies of Robotic Pylorus-Preserving Pancreaticoduodenectomy Using the da Vinci SP (Single Port) System

**DOI:** 10.3390/jcm14093193

**Published:** 2025-05-05

**Authors:** Hyung Sun Kim, Jin Hong Lim

**Affiliations:** Division of Hepatobiliary and Pancreatic Surgery, Department of Surgery, Gangnam Severance Hospital, Yonsei University College of Medicine, Eunju-ro 63-gil, Gangnam-gu, Seoul 06229, Republic of Korea; milk8508@yuhs.ac

**Keywords:** robotic surgery, pylorus-preserving pancreaticoduodenectomy, da Vinci SP system

## Abstract

**Background:** Robot-assisted pylorus-preserving pancreaticoduodenectomy (RPPPD) has been increasingly adopted, leveraging the advantages of robotic technology. RPPPD is rarely performed using the da Vinci SP system. In this study, we address the technical issues encountered during the early experiences with robotic pylorus-preserving pancreaticoduodenectomy (RPPPD) using the da SP Vinci system and propose effective solutions. **Method:** We retrospectively analyzed the outcomes of seven patients who underwent RPPPD using the da Vinci SP system. The primary technical challenges included limited instrument maneuverability, difficulty in maintaining clear surgical views, and the need for precise anastomosis. **Results:** Postoperatively, all patients were discharged without significant complications, with no clinically relevant pancreatic fistulas observed. Only minimal scarring was observed postoperatively. In addition, our results showed that operative time gradually decreased. The operation time was significantly shorter in the RPPPD using the SP system group compared to the RPPPD using the multiport system group. **Conclusions:** Implementing enhanced preoperative planning, advanced intraoperative imaging, and specialized robotic tools can significantly improve surgical efficiency and patient outcomes.

## 1. Introduction

Pancreaticoduodenectomy (Whipple procedure) is a critical surgical intervention used to treat various pancreatic and periampullary diseases. Since the early 2000s, robot-assisted pylorus-preserving pancreaticoduodenectomy (RPPPD) has been increasingly adopted, leveraging the advantages of robotic technology, such as enhanced precision, dexterity, and superior visualization [1,2,3,4,5,6,7]. Among robotic platforms, the da Vinci SP (Single Port) system represents a significant technological advancement, designed to deliver all necessary instruments and cameras through a single 2.5 cm port. The SP system features a fully wristed, elbowed, and flexible instrument set integrated with a 3D high-definition camera. Unlike traditional multiport systems, the SP platform allows for minimal invasiveness with reduced port trauma and improved cosmesis. The system also offers 360-degree adjustability, enhancing the ability to approach deep and narrow anatomical spaces with precision.

Despite these benefits, the use of the da Vinci SP system (Single port system) in complex pancreatic surgeries remains underexplored.

The da Vinci SP system has been adopted across various surgical disciplines, including urologic surgeries (e.g., prostatectomy), gynecologic surgeries (e.g., hysterectomy), colorectal resections, and upper gastrointestinal procedures [8,9,10,11,12]. However, its application in complex pancreatectomy, such as RPPPD, remains limited and underreported. Due to the technical demands of RPPPD, including complex anastomoses and deep retroperitoneal dissection, evaluating the feasibility and safety of SP-based approaches is of clinical importance.

While robotic-assisted pancreaticoduodenectomy (RPD) has demonstrated oncologic safety comparable to open procedures, its application in oncologic pancreatic surgery still requires further investigation. Our study aims to evaluate the technical feasibility, perioperative outcomes, and potential implications for cancer treatment in patients undergoing RPPPD using the da Vinci SP system. The da Vinci SP system represents a significant advancement in this field, offering a single-port approach that minimizes invasiveness while maintaining the benefits of robotic surgery. Despite its minimally invasive advantages, the da Vinci SP system is a relatively new technology, and its use in complex surgeries has not been widely reported [8,9,10,11,12]. Consequently, RPPPD is rarely performed using the da Vinci SP system.

**Figure 1:**
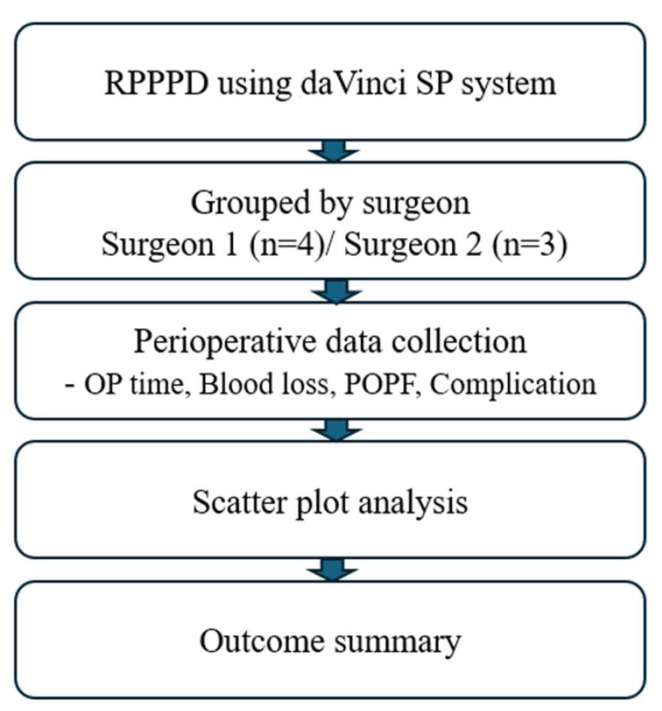
Study flow diagram.

This study aimed to evaluate the early experience and technical feasibility of RPPPD using the da Vinci SP system. By identifying key intraoperative challenges and proposing assistant strategies and workflow modifications, we provide valuable insights for institutions seeking to adopt this technology. A study design flow diagram is presented in Figure 1 to outline the patient selection and evaluation process.

## 2. Materials and Methods

### 2.1. Study Participants and Design

We conducted a retrospective study using data from seven patients with RPPPD using da Vinci SP system at Gangnam Severance Hospital from November 2023 to July 2024. Inclusion criteria included (1) diagnosis of periampullary malignancies amenable to pylorus—preserving pancreaticoduodenectomy, (2) no prior major upper abdominal surgeries, and (3) no evidence of distant metastasis. Patients requiring conversion to open surgery or laparoscopic resection were excluded. We analyzed patients’ postoperative outcomes. All patients who agreed to participate in the study signed an informed consent form before enrollment. The study was conducted in accordance with the Declaration of Helsinki and approved by the Institutional Review Board (IRB) of Gangnam Severance Hospital (3-2024-0423).

### 2.2. Surgical Techniques and Technical Modifications

Surgical procedures were conducted using a single-port system with additional ports to facilitate instrument maneuverability.

**Figure 2:**
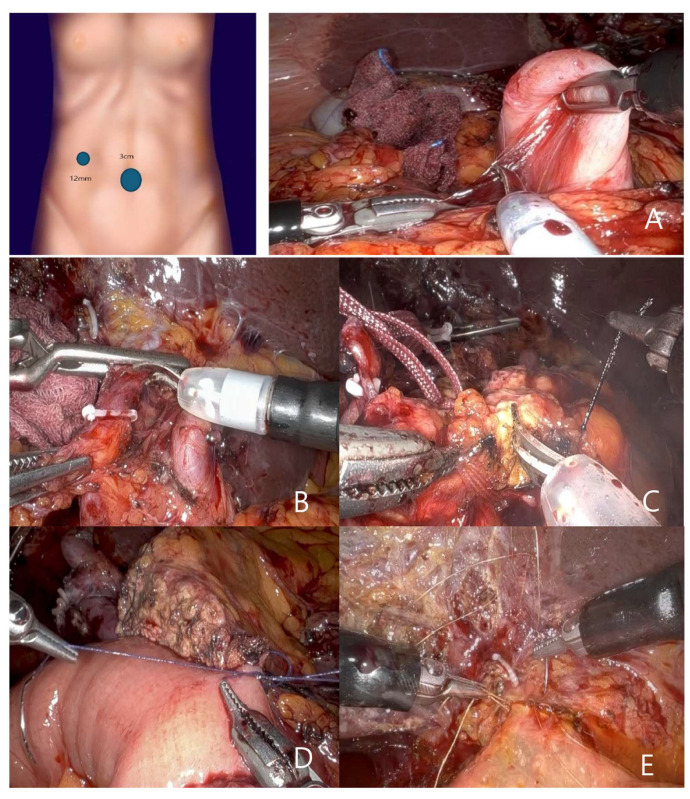
(**A**) By surgeon 1, surgery was performed using a single port with an additional 12 mm port. The primary instruments used were Cadiere forceps and two Maryland bipolar forceps, with scissors. (**B**) Following the transection of the gastroduodenal artery and common bile ducts, (**C**) the pancreas was transected using monopolar scissors (**D**,**E**). Pancreaticojejunostomy (PJ) and choledochojejunostomy (CJ) were performed using a needle holder with Prolene 5-0 for PJ and Monosyn 6-0 for CJ.

For the RPPPD performed by surgeon 1, surgery was performed using a single port with an additional 12 mm port. The 12 mm port facilitated the use of a GIA stapler, suction, gauze insertion and removal, and provided traction with the assistance of an additional surgeon. The primary instruments used were Cadiere forceps and two Maryland bipolar forceps, with scissors used as necessary (Figure 2A).

The procedure began by detaching the omentum from the head of the pancreas. Subsequently, the soft tissue surrounding the hepatoduodenal ligament was dissected. Following the transection of the gastroduodenal artery and common bile ducts (Figure 2B), the first portion of the duodenum was separated using a handheld GIA stapler. Tunneling beneath the neck of the pancreas was accomplished using the Maryland bipolar and Cadiere forceps, and the pancreas was transected using monopolar scissors (Figure 2C).

The second portion of the duodenum was detached from the vena cava. The small bowel mesentery, located on the left side of the ligament of Treitz, was coagulated using Maryland bipolar forceps. A handheld GIA stapler was used to transect the small bowel. After repositioning of the transected small bowel to the right of the ligament of Treitz, retroperitoneal dissection of the pancreas was performed using Maryland bipolar forceps. After completion of the resection, the specimen was placed in a sterile retrieval bag and extracted through the umbilical single-port site.

Pancreaticojejunostomy (PJ) and choledochojejunostomy (CJ) were performed using a needle holder with Prolene 5-0 for PJ and Monosyn 6-0 for CJ (Figure 2D,E).

The pancreaticojejunostomy was performed with continuous suturing of both the anterior and posterior walls using Vicryl 4-0. Duct-to-mucosa anastomosis was completed with approximately six interrupted sutures using 5-0 Prolene.

Hepaticojejunostomy was performed using Monosyn 6-0 sutures, with interrupted sutures on the anterior wall and continuous suturing on the posterior wall.

**Figure 3:**
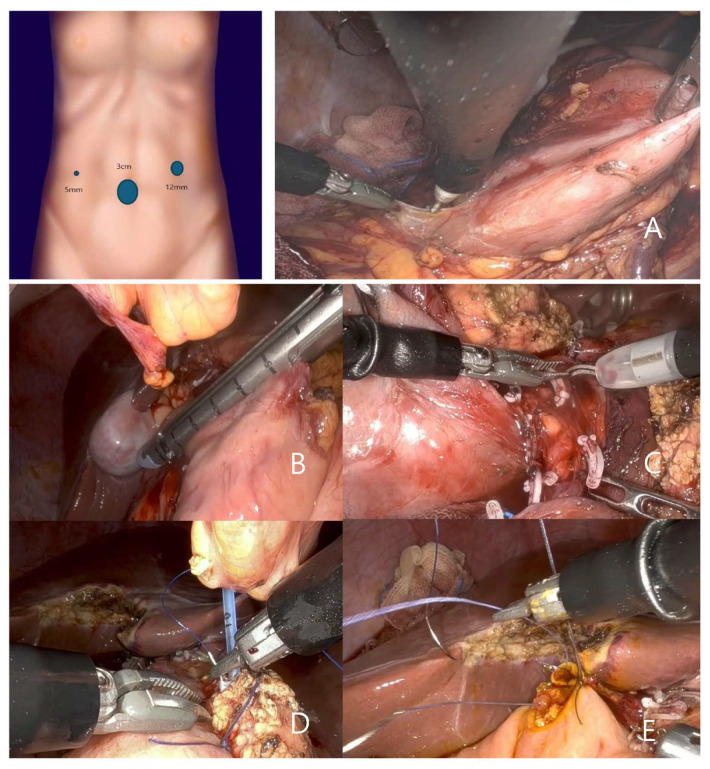
(**A**) By surgeon 2, surgery was performed using a single port with additional 12 mm and 5 mm ports. The primary instruments used were Cadiere forceps and two Maryland bipolar forceps, with cautery hook. (**B**) The first portion of the duodenum was separated using a handheld GIA stapler; (**C**) retroperitoneal dissection of the pancreas was performed using Maryland bipolar forceps, clipping, and a monopolar cautery hook; (**D**,**E**) pancreaticojejunostomy (PJ) and choledochojejunostomy (CJ) were performed using a needle holder with Prolene 5-0 for PJ and Vicryl 4-0 for CJ.

For the RPPPD performed by surgeon 2, surgery was performed using a single port with additional 12 mm and 5 mm ports. The 12 mm port facilitated the use of a GIA stapler, suction, and gauze insertion and removal, while the 5 mm port facilitated the use of traction with the assistance of an additional surgeon. The primary instruments used were Cadiere forceps, Maryland bipolar forceps, and a cautery hook, with scissors used as necessary (Figure 3A).

The procedure began by performing partial omentectomy and resection of the duodenum. Following transection of the right gastric and gastroepiploic arteries, the first portion of the duodenum was separated using a handheld GIA stapler (Figure 3B). Following the transection of the gastroduodenal artery, the soft tissues and lymph nodes surrounding the hepatoduodenal ligament and common bile duct were dissected.

Tunneling beneath the neck of the pancreas was performed using Maryland bipolar forceps and a cautery hook, and the pancreas was transected using monopolar scissors.

Duodenal kocherization was performed. A handheld GIA stapler was used to transect the small bowel. After repositioning of the transected small bowel to the right of the ligament of Treitz, retroperitoneal dissection of the pancreas was performed using Maryland bipolar forceps, clipping, and a monopolar cautery hook. An assistant surgeon performed clipping using an additional port (Figure 3C).

After completion of the resection, the specimen was placed in a sterile retrieval bag and extracted through the umbilical single-port site.

Pancreaticojejunostomy (PJ) and choledochojejunostomy (CJ) were performed using a needle holder with Prolene 5-0 for PJ and Vicryl 4-0 for CJ (Figure 3D,E). The pancreaticojejunostomy was performed with continuous suturing of both the anterior and posterior walls using Vicryl 4-0. Duct-to-mucosa anastomosis was completed with approximately six interrupted sutures using 5-0 Prolene.

Hepaticojejunostomy was performed using Vicryl 4-0 sutures, with interrupted sutures on the anterior wall and posterior wall.

One of the key technical challenges encountered was the limited range of motion due to confined space. To overcome this, we implemented a modified trocar placement strategy, adjusting the docking angles to enhance surgical visibility.

Surgeon 1 positioned the camera from a superior angle, while Surgeon 2 used an inferior approach. The camera angle was adjusted as needed during the procedure to optimize the operative view.

Additionally, instrument exchanges in the SP system were optimized by predefining instrument sequencing, reducing operative delays. These modifications improved our ability to perform precise anastomosis and ensured efficient surgical workflow.

## 3. Results

### 3.1. Clinical Characteristics of Patients with RPPPD Using da Vinci SP System

Table 1 presents the characteristics of the seven patients. In the surgeon 1 group, the age distribution of patients ranged from 55 to 82 years, while the body mass index (BMI) ranged from 21 to 28 kg/m^2^. The group consisted of three men and one woman, none of whom had a prior history of abdominal surgery. The surgeon 2 group comprised three women, aged 45 years and 60 years. Body mass index (BMI) ranged from 17 to 23 kg/m^2^. Additionally, neither patient had a prior history of abdominal surgery.

### 3.2. Perioperative and Postoperative Outcomes of Patients with RPPPD Using da Vinci SP System

In the surgeon 1 group, the operative time ranged from 280 to 520 min, with blood loss ranging from 500 to 800 mL. Postoperatively, all patients were discharged without significant complications, with two patients being classified as having postoperative pancreatic fistula (POPF) Grade A (biochemical leak).

Only two short scars remained, one from the single port incision and one from the additional port incision.

**Figure 4:**
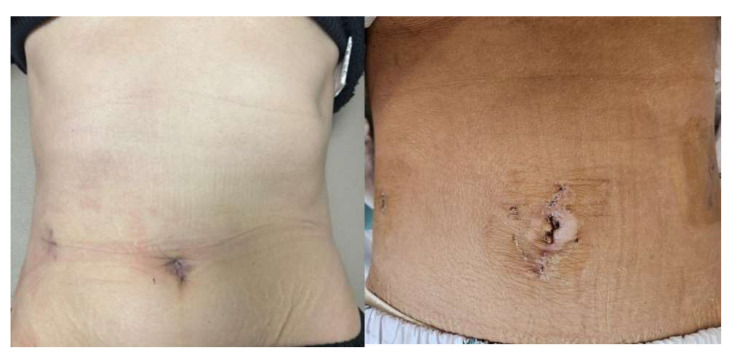
Postoperative wound of patient who underwent surgery by surgeon 2.

In the surgeon 2 group, the operative time varied from 450 to 700 min, with blood loss ranging from 700 to 1200 mL. Postoperatively, one patient had a drain-site infection, which was controlled with antibiotics. Two patients were classified as having POPF Biochemical leak (Table 2). Three short scars remained, one from the single-port incision and two from the additional port incisions (Figure 4).

None required further intervention, and all resolved conservatively within postoperative days 7–14. None of the patients developed delayed gastric emptying symptoms. All anastomoses (pancreas) were pancreatico-jejunal.

**Figure 5:**
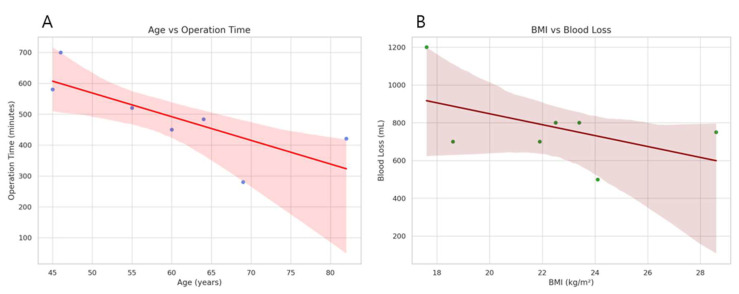
(**A**) Correlation between patient age and operation time. (**B**) Correlation between body mass index (BMI) and intraoperative blood loss.

To evaluate potential predictors of operative complexity, we analyzed correlations between clinical parameters and intraoperative outcomes. Scatter plots revealed a moderate association between higher age and prolonged operative time, as well as lower BMI and greater blood loss. Although a positive trend was observed, the association was not statistically significant (*p* = 0.16; *p* = 0.39). These relationships are visually demonstrated in the scatter plots in Figure 5.

### 3.3. Conparison of Clinical Outcomes Between Multiport RPPPD and RPPPD Using da Vinci SP System

A total of 15 patients were included, with 8 patients undergoing multiport robotic Pancreaticoduodenectomy and 7 patients undergoing single-port (SP) robotic pancreaticoduodenectomy. We compared the outcomes of multiport robotic PPPD and SP robotic PPPD performed during the same period (Table 3).

There were no significant differences in age (54.1 ± 17.5 vs. 60.1 ± 13.1 years, *p* = 0.46) or BMI (22.9 ± 3.7 vs. 22.4 ± 3.7 kg/m^2^, *p* = 0.78) between the two groups.

The operation time was significantly shorter in the single-port group compared to the multiport group (490.7 ± 131.4 vs. 674.9 ± 133.4 min, *p* = 0.02).

There were no statistically significant differences in estimated blood loss (778.6 ± 211.9 vs. 993.8 ± 865.4 mL, *p* = 0.51) or hospital stay (16.3 ± 5.9 vs. 13.5 ± 6.1 days, *p* = 0.38).

The incidence of postoperative pancreatic fistula (POPF) grade and delayed gastric emptying (DGE) did not differ significantly between the multiport and single-port (SP) groups (*p* = 0.80 and *p* = 0.24). There were no severe complications observed in either the multiport or single-port (SP) robotic surgery groups.

## 4. Discussion

Robot-assisted minimally invasive surgery has emerged as a novel method that maintains the advantages of minimally invasive surgery by increasing maneuverability with wristed movement, providing more precise manipulation, and improving visualization in three dimensions [13]. The da Vinci SP platform enables single-port robotic surgery with optional additional ports, facilitating access to complex anatomical regions with minimal invasiveness.

da Vinci SP provides you with the ability to deliver robotic-assisted surgery through a single port. The system’s instruments are optimized for single-port surgery, and advanced vision can help empower surgeons to perform procedures with a range of complexity. The da Vinci SP system offers several advantages and disadvantages, particularly in PPPD.

We have now listed the five major technical challenges and advantages: (1) Performing safe and effective dissection using only monopolar cautery hook and bipolar forceps without the availability of advanced energy devices. (2) Achieving optimal visualization through a 360-degree camera rotation mode, which has a great advantage compared to the Xi system in deep and narrow anatomical spaces. (3) Maintaining minimal invasiveness by utilizing only one or two additional ports beyond the main single-port access. (4) Enabling precise anastomosis with wristed instrument articulation and allowing fine movements and enhanced suturing control. (5) Operating in confined spaces while still accommodating the camera and three fully functional robotic instruments simultaneously.

Compared to traditional open or multi-port robotic approaches, our early experience suggests that SP-RPPPD minimizes trauma while maintaining oncologic safety.

This is particularly beneficial for the delicate and complex surgical maneuvers required in RPPPD, as the added flexibility and control help surgeons navigate intricate anatomical structures more effectively, thus reducing the risk of damage to the surrounding tissues. In addition, the system allows for single-port access with the option to use one or two additional ports, enhancing flexibility for surgeons and optimizing the surgical approaches. This flexibility optimizes the operating field and instrument reach, enhances the overall efficiency of the procedure, and simplifies the handling of complex surgical tasks. However, adapting to the new da Vinci SP system takes time. Nonetheless, surgeon 2 was able to quickly adapt to the da Vinci SP system by performing approximately 10 cholecystectomies with reduced operation time. Subsequently, the surgeon was able to perform complex operations, such as RPPPD. The SP system has the advantage of a short learning curve because it has only one robotic arm that needs to be docked, so even beginners in robotic surgery can easily adapt to the system, making complex surgeries possible.

Recently, it was reported that prostatectomy using a single-port (SP) system has more advantages than surgery performed using a multiport system. The SP system was associated with a lower risk of severe complications as the comorbidity score for robotic surgery increased compared to the multi-port system [8].

In particular, PPPD surgery requires multi-organ resection, multi-vascular resection, and multi-organ anastomosis, and there is a difference in the risk of complications depending on the stability of these techniques. The instrument flexibility of the SP system is suitable for clearly ligating small and fine blood vessels and is suitable for a more precise technique for fine anastomosis, such as pancreaticojejunostomy.

Although the use of one or two additional ports may appear to compromise the concept of single-port surgery, the da Vinci SP system still offers meaningful advantages over conventional multiport approaches. By reducing the total number of incisions by two to three compared to multiport surgery, it minimizes overall surgical trauma. Furthermore, the SP system enables precise dissection and suturing even within narrow anatomical spaces, maintaining both the minimally invasive nature and technical benefits of single-port access.

In our study, all patients were discharged without severe complications and with minimal scarring. Therefore, our approach contributed to the improvement in the quality of life of our patients after surgery. Compared to conventional multiport RPD or open pancreaticoduodenectomy, the operative times and blood loss in our cohort were comparable or favorable, despite the learning curve. Previous reports indicate that traditional RPD typically requires 510–600 min of operative time and involves blood loss exceeding 1000 mL [14,15,16,17], while our SP cohort showed operative times ranging from 280 to 700 min and blood loss of 500–1200 mL, depending on port configuration and surgical complexity. In our institutional experiences with multiport robotic PPPD, the operative time ranged from 540 to 900 min, and estimated blood loss ranged from 700 to 2000 mL.

The operation time was significantly shorter in the single-port group compared to the multiport group (*p* = 0.02). Although the difference was not statistically significant, the lower incidence of delayed gastric emptying in the single-port (SP) group may be attributed to reduced postoperative pain, facilitating earlier mobilization and gastrointestinal recovery.

Compared to our institutional data on multiport robotic PPPD, the PPPD cases using the SP system in this study demonstrated comparable or reduced intraoperative burden.

To evaluate potential predictors of operative complexity, we analyzed correlations between clinical parameters and intraoperative outcomes. Scatter plots revealed a moderate association between higher age and prolonged operative time, as well as lower BMI and greater blood loss. These relationships are visually demonstrated in the scatter and box plots in Figure 5.

Nevertheless, the system has disadvantages, such as the lack of dedicated energy devices, which limits the available tools for dissection and hemostasis, potentially complicating the procedure. Surgeons may have to rely on less optimal instruments, which affects the precision and speed of surgical tasks. Instead of an energy device, we used Maryland bipolar forceps and a monopolar cautery hook. The coagulation energy exceeded expectations, while the bipolar grasping force adequately compensated for the lack of a dedicated energy device.

Another disadvantage is the time-consuming process of changing instruments in the SP system compared with multiport systems, which can extend operative times and increase the complexity of surgery. Frequent instrument swaps can disrupt the flow of the procedure, leading to a longer surgical duration and increased patient exposure to anesthesia. However, in the SP system, docking-requiring port placement only needs to be performed once, thus reducing time compared with that required for the Xi system.

Despite these challenges, early case reports demonstrated that RPPPD is technically feasible with acceptable operative times and surgical outcomes. Multi-institutional studies have indicated that RPPPD may result in reduced intraoperative blood loss and shortened hospital stays compared with those in traditional approaches [14,15,16,17,18,19,20,21,22]. Our initial experience with the da Vinci SP system shows promise, particularly in terms of reduced invasiveness and enhanced precision in surgical tasks.

Continuous technological advancements in robotic systems have improved the efficiency and safety of RPPPD, thereby contributing to enhanced recovery and reduced complication rates [23,24,25,26]. Our findings suggest that minimally invasive RPPPD may contribute to improved postoperative recovery and reduced complications, which are crucial for oncologic patients. In addition, the patients’ level of postoperative pain was lower compared to patients who underwent open surgery. Although statistical analysis was not possible due to the small number of patients, these results can be used as a basis for future research. While our study does not directly assess long-term oncologic outcomes, prior studies have demonstrated that minimally invasive approaches, including robotic surgery, may lead to reduced postoperative complications, potentially impacting cancer recurrence rates and long-term survival.

This study has several limitations. The number of patients is still small. It is not possible to compare it with other surgeries, so it is not possible to describe the clear advantages. Also, because the surgeries were performed recently, the long-term outcomes of the patients have not yet been described.

Future research should focus on comparative oncologic outcomes, recurrence rates, and long-term quality of life between SP-robotic, multi-port robotic, and open approaches.

By leveraging its advantages, such as wrist articulation and flexible port management, and addressing its limitations, such as the lack of dedicated energy devices and time-consuming instrument exchanges, surgeons can optimize surgical outcomes.

## 5. Conclusions

Ongoing innovations and positive initial experiences with the da Vinci SP system highlight its potential for advancing RPPPD.

## Figures and Tables

**Table 1:** Clinical characteristics of patients with RPPPD using da Vinci SP system.

	**Surgeon 1**				**Surgeon 2**		
	**Patient 1**	**Patient 2**	**Patient 3**	**Patient 4**	**Patient 5**	**Patient 6**	**Patient 7**
Age (years)	64	82	69	55	46	45	60
Sex	Female	Male	Male	Male	Female	Female	Female
BMI (kg/m^2^)	24.1	21.9	22.5	28.6	17.6	18.6	23.4
Medical hx	None	None	None	None	None	None	None
Surgical hx	None	None	None	None	None	None	None

hx, History; BMI, Body mass index.

**Table 2:** Perioperative and postoperative outcomes of patients with RPPPD using da Vinci SP system.

	**Surgeon 1**				**Surgeon 2**		
	**Patient 1**	**Patient 2**	**Patient 3**	**Patient 4**	**Patient 5**	**Patient** **6**	**Patient 7**
Operation time (min)	484	421	280	520	700	580	450
Blood loss (mL)	500	700	800	750	1200	700	800
Complication	None	None	None	None	Drain site infection	None	None
POPF	None	Biochemical leak	None	Biochemical leak	None	Biochemical leak	Biochemical leak
Discharge (d)	13	26	9	11	21	16	18
Clinical diagnosis	SCN	AOV cancer(cTxN0)	Pancreatic cancer (cT2N+)	Pancreatic cancer (cT2N+)	CBD cancer (cTxN0)	SPN	Pancreatic cancer (cT3N0)
Pathology	SCN [2.2 × 1.3 cm, LN(0/12)]	AOV cancer [T1aN0, 0.2 × 0.2 cm,WD, LN(0/13)] RM(negative)	Pancreatic cancer [T2N2, 3.5 × 2.8 cm, MD,LN(4/24)] RM(negative)	Pancreaticancer [T3N2, 4.2 × 4.1 cm, MD, LN(5/19)] RM(negative)	CBD cancer [T2N1, 3.2 × 2.2 cm, MD, LN(1/42)] RM(negative)	SPN [3.2 × 2.2 cm, LN(0/16)]	Pancreatic cancer [T3N0, 4.1 × 2.3 cm, LN(0/48)] RM(negative)

POPF, Postoperative pancreatic fistula; SCN, Serous cystic adenoma; AOV, Ampulla of Vater; CBD, Common bile duct; SPN, Solid pseudopapillary neoplasm; WD, Well differentiation; MD, Moderately differentiation; LN (number/number), Lymph node (Number of metastatic carcinoma/Total number of harvested lymph node); RM, resection margin.

**Table 3:** Comparison between multiport and single-port (SP) Robotic PPPD.

**Variable**		**Mean ±** **SD, or *n* (%)**		***p*-Value**
		**Multiport (*n* = 8)**	**Single Port (*n* = 7)**	
Age (years)		54.1 ± 17.5	60.1 ± 13.1	0.46
Sex	Male	4 (50%)	3 (42.9%)	1.00
	Female	4 (50%)	4 (58%)	
BMI (kg/m^2^)		22.9 ± 3.7	22.4 ± 3.7	0.78
Diganosis	Benign	1 (12.5%)	2 (28.6%)	0.89
	Malignancy	7 (87.5%)	5 (71.4%)	
Operation time (min)		674.9 ± 133.4	490.7 ± 131.4	0.02
Estimated blood loss (mL)		993.8 ± 865.4	778.6 ± 211.9	0.51
Hospital stay (days)		13.5 ± 6.1	16.3 ± 5.9	0.38
POPF	None	5 (62.5%)	3 (42.9%)	0.80
	Biochemical leak	3 (37.5%)	4 (57.1%)	
	B&C	0	0	
Delayed gastric emptyng	None	5 (62.5%)	7 (100%)	0.24
	DGE A	3 (37.5%)	0 (0%)	
	DGE B&C	0 (0%)	0 (0%)	
Complication Grade ≥ 3		0 (0%)	0 (0%)	1.00

POPF, Postoperative pancreatic fistula; SD, Standard deviation; DGE, Delayed gastric emptying.

## Data Availability

The original contributions presented in this study are included in the article. Further inquiries can be directed to the corresponding author.

## Abbreviations

The following abbreviations are used in this manuscript:

RPPPD	Robotic pylorus-preserving pancreaticoduodenectomy
BMI	Body mass index
POPF	Postoperative pancreatic fistula
SCN	Serous cystic adenoma
AOV	Ampulla of Vater
CBD	Common bile duct
SPN	Solid pseudopapillary neoplasm
